# Separation and Characterization of Therapeutic Oligonucleotide Isomer Impurities by Cyclic Ion Mobility Mass Spectrometry

**DOI:** 10.1021/jasms.4c00197

**Published:** 2024-07-31

**Authors:** Shogo Omuro, Takao Yamaguchi, Taiji Kawase, Kenji Hirose, Tokuyuki Yoshida, Takao Inoue, Satoshi Obika

**Affiliations:** † Graduate School of Pharmaceutical Sciences, Osaka University, 1-6 Yamadaoka, Suita, Osaka 565-0871, Japan; ‡ Nihon Waters KK, Kitashinagawa, Shinagawa, Tokyo 140-0001, Japan; § Division of Molecular Target and Gene Therapy Products, National Institute of Health Sciences, 3-25-26 Tonomachi, Kawasaki-ku, Kawasaki, Kanagawa 210-9501, Japan

## Abstract

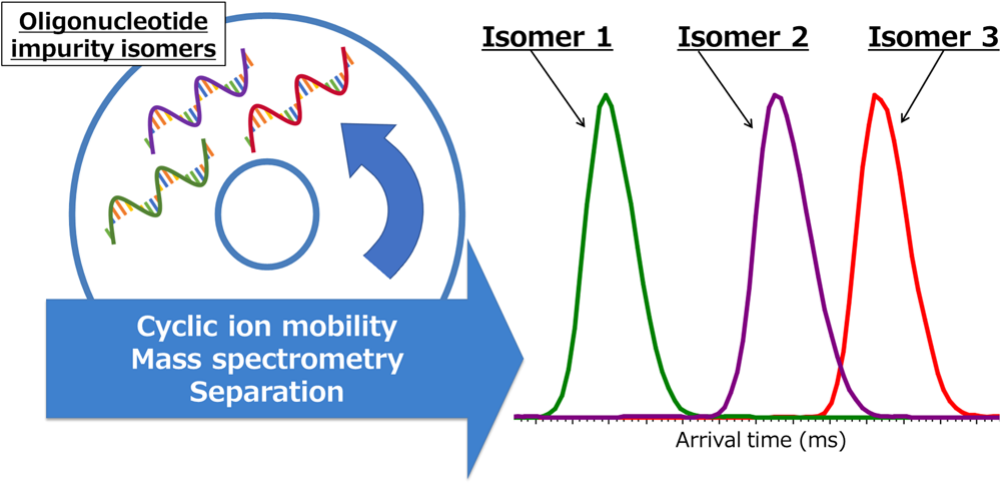

Therapeutic oligonucleotides such as antisense oligonucleotide (ASO) and small interfering RNA (siRNA) are among the most remarkable modalities in modern medicine. ASOs and siRNA are composed of single- or double-stranded 15–25 mer synthesized oligonucleotides, which can be used to modulate gene expression. Liquid chromatography–mass spectrometry (LC/MS) is a necessary technique for the quality control of therapeutic oligonucleotides; it is used to evaluate the quantities of target oligonucleotides and their impurities. The widely applied oligonucleotide therapeutic quantitation method uses both ultraviolet (UV) absorbance and the MS signal intensity. Peaks separated from the main peak, which contains full-length product, are generally quantitated by UV. However, coeluting impurities, such as *n* – 1 shortmers, abasic oligonucleotides, and PS → PO (phosphorothiate to phosphodiester) oligonucleotides, are quantitated by MS. These coeluting impurities can also be comprised of various isomers with the same modification, thus increasing the difficulty in their separation and relative quantitation by LC/MS. It is possible that a specific isomer with a certain structural form induces toxicities. Therefore, characterization of each isomer separation is in high demand. In this study, we separated and characterized oligonucleotide isomers by employing a cyclic ion mobility mass spectrometry (cyclic IMS) system, which allows the separation of ions with the same *m*/*z* ratio based on their structural differences. Patisiran antisense and sense strands and their *n* – 1 and abasic isomers were used as sample sequences, and their ratio characterization was achieved by cyclic IMS. In addition, we evaluated the PS → PO conversion isomers of the antisense strand of givosiran, which originally contained four PS modification sites. The PS → PO isomers exhibited specific and distinguishable mobiligram patterns. We believe that cyclic IMS is a promising method for evaluating therapeutic oligonucleotide isomers.

## Introduction

**Figure 1:**
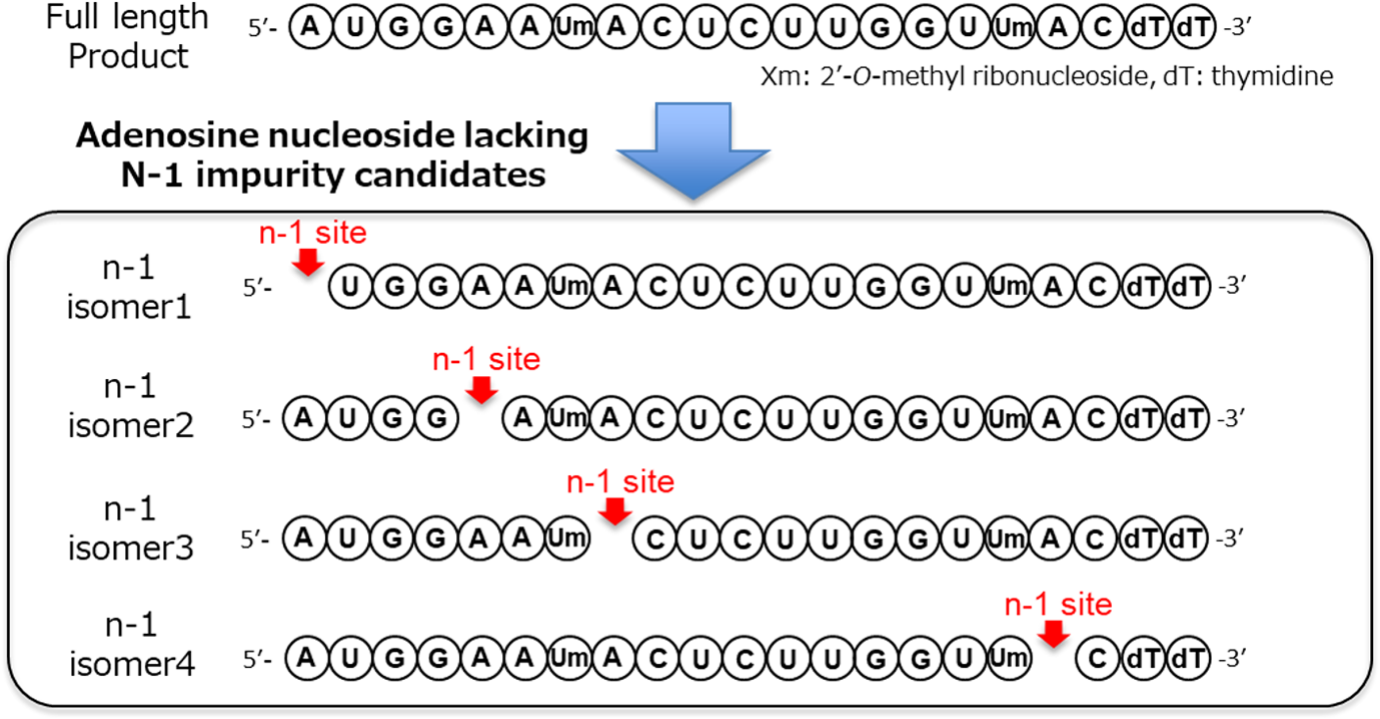
Patisiran antisense strand and its potential *n* – 1 impurity isomers.

In recent times, drug candidate modalities have been rapidly diversifying and increasing in complexity. Antisense oligonucleotides (ASO) and small interfering RNAs (siRNA) are among the most significant therapeutic oligonucleotides, and more than 10 such drug products have been approved in the USA, the EU, and/or Japan in recent years.^1,2^ ASO and siRNA are single- or double-stranded chemically synthesized oligonucleotides that can be used to modulate gene expression. In many cases, impurities of therapeutic oligonucleotides have a similar physicochemical property as full-length product (FLP); therefore, it is difficult to separate using LC/UV. Therefore, LC/UV/MS plays an important role in the analysis of therapeutic oligonucleotide impurities to achieve the separation.^3−7^ Impurities known to coelute with the FLP include the PS → PO conversion products, in which the phosphorothioate (PS) linkage is replaced by a phosphodiester (PO) linkage; abasic impurities, which lack one or more purine nucleobases; and *n* – 1 impurities, which are produced owing to the deletion of a single nucleotide (Figures S1A–S1C).^8−10^ Here, the advantage of using LC/MS is the separation capability of coeluting impurities by their *m*/*z* difference. However, even in the method of evaluating impurities as an impurity group from *m*/*z* information acquired by MS, it is difficult to characterize which part of the nucleotide sequence has been changed and at what ratio. The tandem mass spectrometry (MS/MS) approach is useful for determining the modification site by collision-induced dissociation fragmentation. However, it is difficult to characterize the ratio of isomer impurities. Here, we explain the use of using the *n* – 1 impurity of the siRNA patisiran antisense strand (sequence: 5′-AUGGAAUmACUCUUGGUUmACdTdT-3′, where Um and dT represent 2′-*O*-methyluridine and thymidine, respectively) as an example.^11^ As shown in Figure 1, if the *n* – 1 impurity lacks an adenosine phosphate molecule, four impurity-structure candidates can be assumed according to the molecular weight information obtained from MS. The mechanism-of-action of siRNA is to suppress the expression of the target protein by recognizing and cleaving the complementary mRNA sequence.^12,13^ Isomer 1 is a shortmer that lacks the 3′-end nucleotide of the original sequence; therefore, it is expected to hybridize to the same target mRNA site as the original sequence. In contrast, isomers 2–4 have an internal *n* – 1 site and hence may give rise to off-target toxicity caused by hybridization with nontarget mRNA sequences. A white paper proposing the impurity control of oligonucleotide therapeutics suggested that internal *n* – 1 compounds pose a higher risk than the terminal *n* – 1 sequences.^8^ In addition to hybridization-dependent off-target toxicities, it is possible that a certain isomer induces hybridization-independent toxicities. Therefore, the estimation of the ratio of these isomer impurities mixtures is demanded for the characterization of therapeutic oligonucleotides.

Cyclic ion mobility mass spectrometry (cyclic IMS) is a gas-phase separation technique coupled with MS, which enables the separation of molecules not only by the *m*/*z* data but also by the differences in the structures of target ions with high resolution. A prominent feature of the cyclic IMS system is that a circular “race-track”-shaped mobility cell is installed orthogonally to the main ion optical axis. For acquiring high-resolution mobiligrams, target ions are passed around multiple times within the circular mobility cell to extend the separation. The separation and characterization results of several significant molecules including peptides, proteins, and oligosaccharides, which are difficult to achieve by LC/MS, were recently obtained using high-resolution cyclic IMS.^14−22^ To the best of our knowledge, the comprehensive isomer separation and characterization analysis of therapeutic oligonucleotides using cyclic IMS has not yet been reported.

In this study, we analyzed structurally different oligonucleotide isomers using cyclic IMS and revealed that abasic and *n* – 1 isomer impurities could be separated. Also, we analyzed the givosiran sequence antisense strand, which originally contained four PS modifications, and its PS → PO impurity isomers. Interestingly, each isomer exhibited an identifiable specific mobiligram. These results indicate that cyclic IMS can be used for the characterization of therapeutic oligonucleotides.

## Experimental Section

### Chemicals and Reagents

Triethylamine (TEA), 1,1,1,3,3,3-hexafluoro-2-propanol (HFIP), and methanol (MeOH) were purchased from Kanto Chemical Co., Inc. (Tokyo, Japan). Purified water was obtained using a Millipore Milli-Q system (Millipore, Billerica, MA). All oligonucleotides were purchased from GeneDesign Inc. (Osaka, Japan) in the form of lyophilized powder. In this study, the dSpacer structure was applied as an abasic model owing to oligonucleotide sequence availability (Figure S1D).

### MS Data Acquisition

The cyclic IMS data were acquired using a Waters SELECT SERIES cyclic IMS system (Milford, MA). The oligonucleotide sequences used in this study are shown in Table 1. The concentration of the oligonucleotides was 0.1 nmol/μL. The oligonucleotide samples were prepared by dissolving them in 30% MeOH containing 10 mM TEA and 25 mM HFIP. Cyclic IMS measurement was performed using the direct infusion method under the following conditions in the negative electrospray ionization (ESI) MS mode: flow injection rate, 20 μL/min; data acquisition rate, 1 Hz (MS scan); and mass range, *m*/*z* 500–2500. All data were acquired for 60 scans, and the Waters MassLynx software was used for data acquisition. The MS conditions were as follows: capillary voltage, 2.0 kV; sampling cone voltage, 50 V; source temperature, 120 °C; desolvation temperature, 300 °C. The IMS conditions were as follows: pushes per bin, 1; TW static height, 22.0 V; helium gas flow rate, 120 mL/min: nitrogen gas flow rate, 40 mL/min. Cyclic IMS multipass data were acquired by manual operation.

**Table 1:** Oligonucleotide Sequences Used in This Study^a^

ID	description	sequence (5′ to 3′)
ON-1	DNA 8 mer spacer position 1	5′- d(STCGATCG)-3′
ON-2	DNA 8 mer spacer position 2	5′- d(ATCGSTCG)-3′
ON-3	DNA 8 mer *n* – 1 position 1	5′- d(TCGATCG)-3′
ON-4	DNA 8 mer *n* – 1 position 2	5′- d(ATCGTCG)-3′
ON-5	DNA random 8 mer spacer position 1	5′- d(STCGATCG)-3′
ON-6	DNA random 8 mer spacer position 2	5′- d(ATCGSTCG)-3′
ON-7	DNA + PS 8 mer spacer position 1	5′- d(S∧T∧C∧G∧A∧T∧C∧G)-3′
ON-8	DNA+ PS 8 mer spacer position 2	5′- d(A∧T∧C∧G∧S∧T∧C∧G)-3′
ref-1^b^	patisiran sense strand FLP	5′- GUmAACmCmAAGAGAGUmAUmUmCmCmAUmdTdT-3′
ON-9	patisiran sense strand *n* – 1 site 1	5′- GUmA_CmCmAAGAGAGUmAUmUmCmCmAUmdTdT-3′
ON-10	patisiran sense strand *n* – 1 site 2	5′- GUmAACmCmA_GAGAGUmAUmUmCmCmAUmdTdT-3′
ON-11	patisiran sense strand *n* – 1 site 3	5′- GUmAACmCmAAGAGAGUmAUmUmCmCm_UmdTdT-3′
ON-12	patisiran sense strand abasic site 1	5′- GUmASCmCmAAGAGAGUmAUmUmCmCmAUmdTdT-3′
ON-13	patisiran sense strand abasic site 2	5′- GUmAACmCmASGAGAGUmAUmUmCmCmAUmdTdT-3′
ON-14	patisiran sense strand abasic site 3	5′- GUmAACmCmAAGAGAGUmAUmUmCmCmSUmdTdT-3′
ref-2^b^	patisiran antisense FLP	5′- AUGGAAUmACUCUUGGUUmACdTdT-3′
ON-15	patisiran antisense strand abasic site 1	5′- AUGGAAUmACUCUUGGUUmSCdTdT-3′
ON-16	patisiran antisense strand abasic site 2	5′- AUGGAAUmSCUCUUGGUUmACdTdT-3′
ON-17	patisiran antisense strand abasic site 3	5′- AUGGSAUmACUCUUGGUUmACdTdT-3′
ON-18	patisiran antisense strand abasic site 4	5′- SUGGAAUmACUCUUGGUUmACdTdT-3′
ON-19	patisiran antisense strand *n* – 1 site 1	5′- AUGGAAUmACUCUUGGUUm_CdTdT-3′
ON-20	patisiran antisense strand *n* – 1 site 2	5′- AUGGAAUm_CUCUUGGUUmACdTdT-3′
ON-21	patisiran antisense strand *n* – 1 site 3	5′- AUGG_AUmACUCUUGGUUmACdTdT-3′
ON-22	patisiran antisense strand *n* – 1 site 4	5′-_UGGAAUmACUCUUGGUUmACdTdT-3′
ON-23	givosiran antisense (PS → PO)1 isomer 1	5′-UmAf^∧^AfGfAmUfGmAfGmAfCmAfCmUfCmUfUmUfCmUfGm^∧^Gm^∧^Um-3′
ON-24	givosiran antisense (PS → PO)1 isomer 2	5′-Um^∧^AfAfGfAmUfGmAfGmAfCmAfCmUfCmUfUmUfCmUfGm^∧^Gm^∧^Um-3′
ON-25	givosiran antisense (PS → PO)1 isomer 3	5′-Um^∧^Af^∧^AfGfAmUfGmAfGmAfCmAfCmUfCmUfUmUfCmUfGmGm^∧^Um-3′
ON-26	givosiran antisense (PS → PO)1 isomer 4	5′-Um^∧^Af^∧^AfGfAmUfGmAfGmAfCmAfCmUfCmUfUmUfCmUfGm^∧^GmUm-3′
ON-27	givosiran antisense (PS → PO)3 isomer 1	5′-Um^∧^AfAfGfAmUfGmAfGmAfCmAfCmUfCmUfUmUfCmUfGmGmUm-3′
ON-28	givosiran antisense (PS → PO)3 isomer 2	5′-UmAf^∧^AfGfAmUfGmAfGmAfCmAfCmUfCmUfUmUfCmUfGmGmUm-3′
ON-29	givosiran antisense (PS → PO)3 isomer 3	5′-UmAfAfGfAmUfGmAfGmAfCmAfCmUfCmUfUmUfCmUfGm^∧^GmUm-3′
ON-30	givosiran antisense (PS → PO)3 isomer 4	5′-UmAfAfGfAmUfGmAfGmAfCmAfCmUfCmUfUmUfCmUfGmGm^∧^Um-3′
ON-31	givosiran antisense FLP	5′-Um^∧^Af^∧^AfGfAmUfGmAfGmAfCmAfCmUfCmUfUmUfCmUfGm^∧^Gm^∧^Um-3′
ON-32	givosiran antisense (PS → PO)4	5′-UmAfAfGfAmUfGmAfGmAfCmAfCmUfCmUfUmUfCmUfGmGmUm-3′

a d(): DNA sequence, S: abasic site, ^∧^: phosphorothioate, _: *n* – 1 site, Xm: 2′-*O*-methyl ribonucleotide, Xf: 2′-F ribonucleoside, dT: thymidine.

b Ref sequences are not used in this study, described for sequence comparison.

Oligonucleotide ions exhibit multiple negative charge distributions; therefore, several abundant charge state oligonucleotide ions were selected and monitored to achieve the best charge state for separation. The resolution of cyclic IMS increased in correlation with the cycle pass time number. However, in the circular mobility cell, the fast-moving ions eventually catch up with the slow-moving ones as the number of passes increases, and the separation is lost (wrap-around).^18,23^ In this study, the ion mobility separation or cycle pass time number was optimized to ensure that wrap around effects were not observed.

## Results and Discussion

### Separation of Short-Chain Oligonucleotides by Cyclic IMS

**Figure 2:**
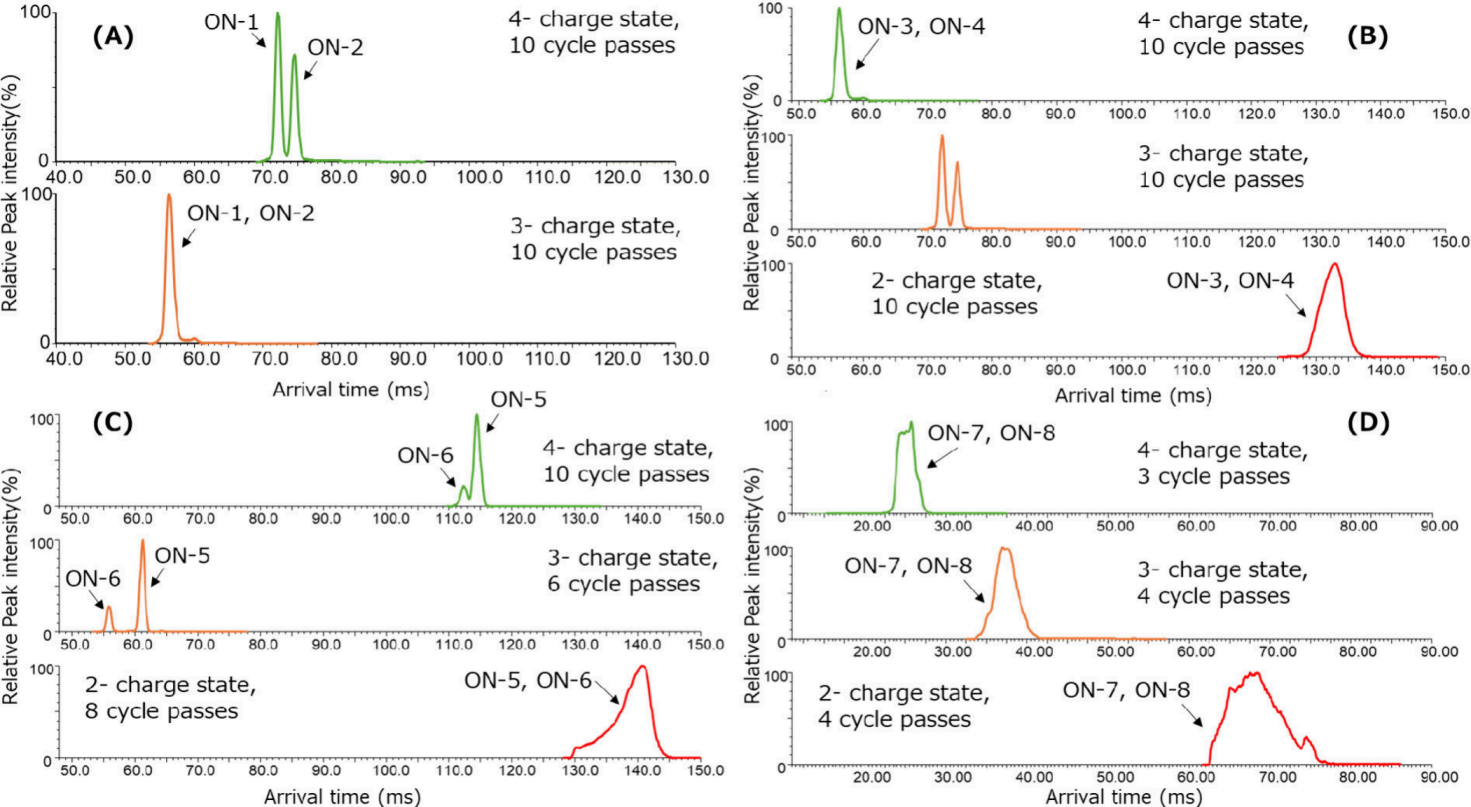
Mobiligrams of the mixtures of same-mass short-chain oligonucleotides: (A) DNA 8 mer abasic isomer sequence mixture (ON-1 and ON-2), (B) DNA 8 mer *n* – 1 isomer sequence mixture (ON-3 and ON-4), (C) DNA random 8 mer abasic isomer sequence mixture (ON-5 and ON-6), and (D) DNA + PS 8 mer abasic isomer sequence mixture (ON-7 and ON-8).

For the feasibility study of therapeutic oligonucleotides cyclic IMS analysis, 7–8 mer short-chain oligonucleotides, including the abasic, *n* – 1, and PS isomer sequences (ON-1–8), were used as samples. Figure 2 shows the cyclic IMS mobiligrams of the 1:1 mixtures of ON-1/ON-2, ON-3/ON-4, ON-5/ON-6, and ON-7/ON-8. As a result of the measurement, the separation of mixed samples was achieved in 3– and 4– charge state measurements, as shown in Figures 2A, 2B, and 2C. Interestingly, the mobiligram separation patterns were different for each of the charge states, which indicates that the optimal charge state for cyclic IMS separation is different for each sample. In contrast, for sequences containing PS modifications, no separation by cyclic IMS was observed under the same conditions that proved successful for the other DNA samples, regardless of charge state ((Figure 2D). The resolution of cyclic IMS can be improved by increasing the circular mobility cell pass through time. However, the PS-modified sequence exhibited broader mobiligram peaks than the corresponding PO sequence and reached the wrap-around at three- or four-times mobility cell laps. As the PS modification replaces a nonbridging oxygen atom with a sulfur atom, the phosphorus atom becomes a chiral center.^24^ The mobiligram of the 8 mer PS-modified oligonucleotide is composed of 128 diastereomers, and the results show the sum of the separated peaks of these diastereomers. This broader mobiligram tendency has also been reported in previous studies.^25,26^

### siRNA Patisiran Impurity Isomer Separation by Cyclic IMS

Based on the above results, we selected the approved full-PO siRNA patisiran sense and antisense strands as models for oligonucleotide isomer separation to confirm the cyclic IMS separation capability. As oligonucleotides exhibit different mobiligram patterns depending on their charge states, we evaluated the mobiligrams of several charge states and selected the most preferable one for separation. To determine the best charge state for the separation of therapeutic oligonucleotide isomers, optimization was conducted as follows. We first confirmed the intensity of each charge state for each isomer. Three to four high-intensity charge states were selected for cyclic ion mobility spectrometry (IMS) separation. We then prepared a mixture of isomers and assessed the separation using cyclic IMS. The number of passes was incrementally increased until reaching the maximum number of passes that avoided “wrap-around.” This process was repeated for each selected high-intensity charge state to identify the optimal charge state for separation.

The mixing ratios of each abasic or *n* – 1 impurity isomer for this study were set to 1:1:1, 3:1:1, 3:3:1, and 1:1:5, and the mobiligram separation patterns were evaluated (e.g., *n* – 1 site 1:*n* – 1 site 2:*n* – 1 site 3 = 1:1:1). These mixing patterns were employed to assess the feasibility of evaluating differences in various ratios for each impurity isomer. The % relative response was defined to determine whether the mixing ratio was reflected in the mobiligram separation patterns. For the calculation of % relative response, the peak intensity of each isomer was measured, and the response factor of each isomer was determined. The evaluated values were calculated using the following formula:


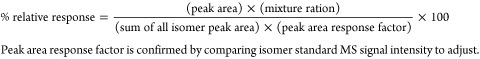


**Figure 3:**
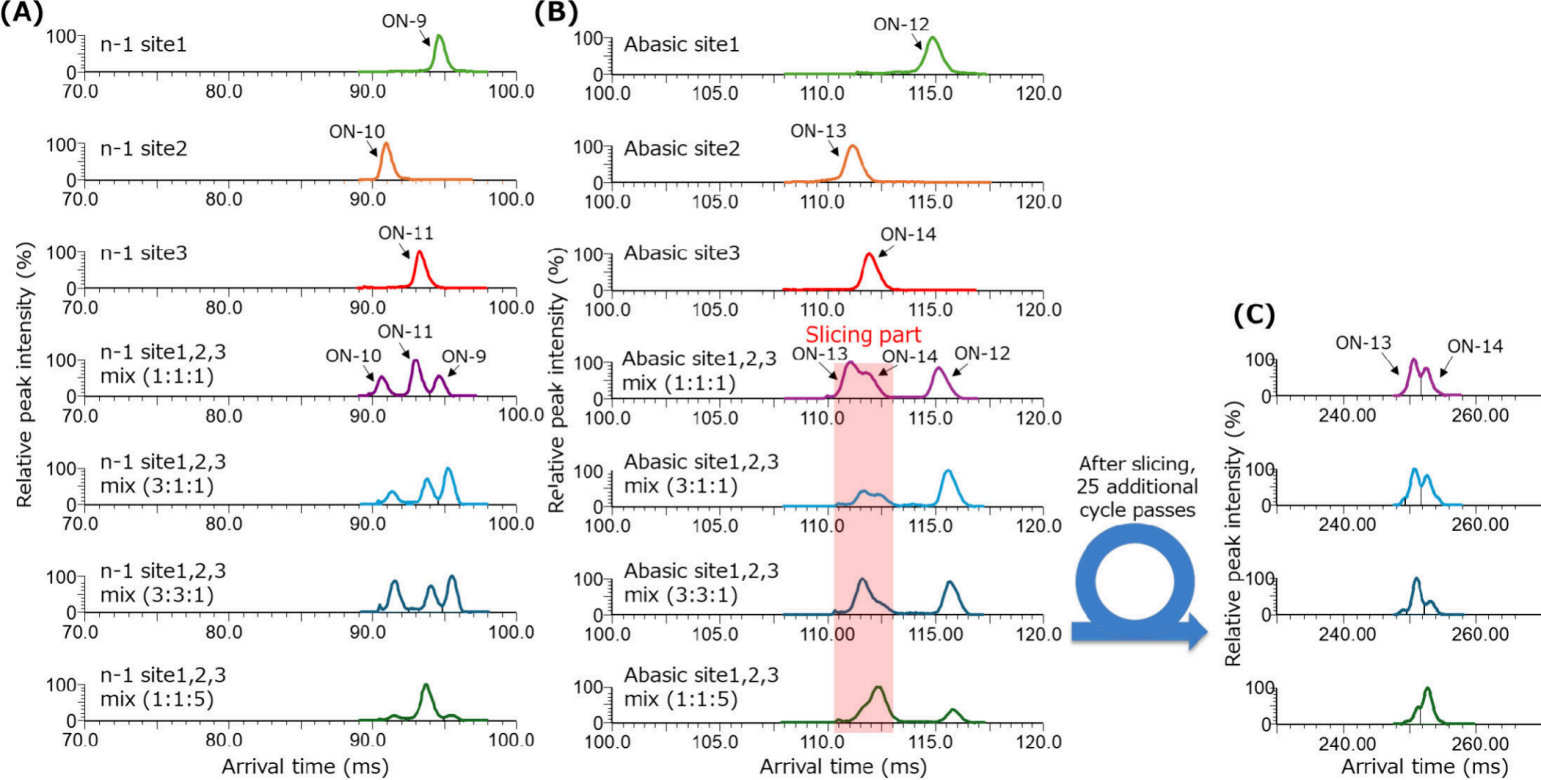
Mobiligrams of the mixtures of *n* – 1 and abasic impurity isomers of the patisiran sense strand: (A) Mobiligrams of the *n* – 1 impurity isomers of the patisiran sense strand acquired at the 10– charge state by 17 cycles passed. (B) Mobiligrams of the abasic impurity isomers of the patisiran sense strand acquired at the 10– charge state by 17 cycles passed. (C) Mobiligrams after slicing applied to the red-colored highlighted part.

The mobiligrams and % relative responses of the *n* – 1 and abasic isomer mixtures of the patisiran sense strand are shown in Figure 3 and Table S1, respectively. For the separation of the mixture of *n* – 1 isomers, a 10– negative charge state and 17-cycle-pass conditions were selected. As shown in Figure 3A, the *n* – 1 isomers of the sense strand were separated. Good % relative responses were observed between 85.4 and 142.0% for the evaluated samples. The evaluated samples were isobaric but different in the location of the *n* −1 site, which appears to influence the peak intensity of the isomers. The peak intensity of the *n* – 1 site 3 was 1.90 times that of the *n* – 1 site 1 and 2.45 times that of *n* – 1 site 2. These results suggest that the mass peak intensity of the oligonucleotide isomers differs, although each of them is expected to have similar physicochemical properties.

Mobiligrams of the mixtures of abasic isomers are shown in Figure 3B and Figure 3C. As with the *n* – 1 isomers, a 10– negative charge state and 17-cycle-pass conditions were applied for the separation. The abasic site 1 peak was separated from the other peaks; however, abasic site 2 and abasic site 3 showed incomplete separation, as shown in Figure 3B. For this reason, the slicing technique was applied to this part, which is a technique to select and put the target ions to cycle pass again and eject the rest of the ions. In this case, 25 additional cycle passes were applied for further separation. The improved mobiligram patterns after slicing are shown in Figure 3C. Even after the slicing, the % relative responses at the mixing ratios of 1:1:1, 3:1:1, and 3:3:1 showed adequate results (Table S1). However, the results for the mixing ratio of 1:1:5 were relatively deviated as follows: abasic site 1: 130.4%, abasic site 2: 166.8%, and abasic site 3: 86.6%. This may have occurred because the 1:1:5 mixing ratio is the most excessive mixture condition that we tested. Moreover, the asymmetrical peak areas obtained after the slicing were vertically separated for area calculation.

**Figure 4:**
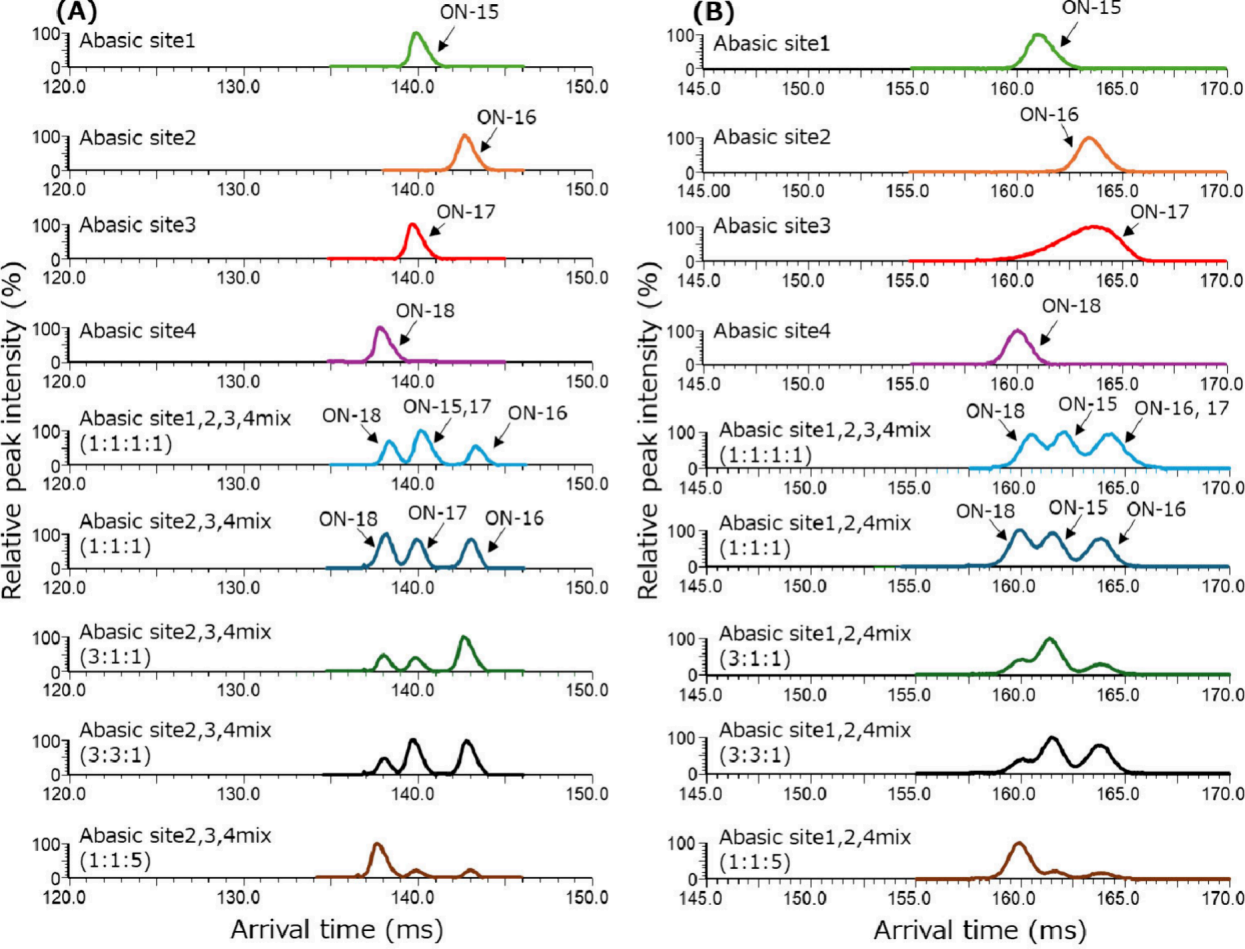
Mobiligrams of the abasic impurity isomers of the patisiran antisense strand: (A) Mobiligrams of patisiran antisense strand abasic impurity isomers acquired at the 9– charge state by 21 cycles passed. (B) Mobiligrams of patisiran antisense strand abasic impurity isomers acquired at the 8– charge state by 20 cycles passed.

Next, we evaluated the separation of four abasic impurity isomers of the patisiran antisense strand (ON-15–18) for further investigation of cyclic IMS separation capability. The mobiligrams of abasic isomers are shown in Figure 4. The 6– to 9– charge state mobiligrams were obtained, but the condition that enables the separation of the four isomers was not determined, as shown in Figure 4A and Figure 4B. However, in the 9– and 8– charge state mobiligrams, complementary separation patterns were obtained as follows: abasic sites 2 and 4 were separated in the 9– charge state, whereas sites 1 and 3 were not separated. Conversely, abasic sites 1 and 4 were separated in the 8– charge state, whereas sites 2 and 3 were not separated.

Here, we focus on the different separation patterns obtained at each charge state. We tried to separate up to three isomer mixtures as follows: abasic sites 2, 3, and 4 at the 9– charge state and abasic sites 1, 2, and 4 at the 8– charge state. The % relative responses for these results are shown in Table S2. The % relative responses at the 8– and 9– charge states were 85.0–116.6% and 81.5–115.5%, respectively. Thus, good % relative responses (between 80% and 120%) were achieved for both charge states.

**Figure 5:**
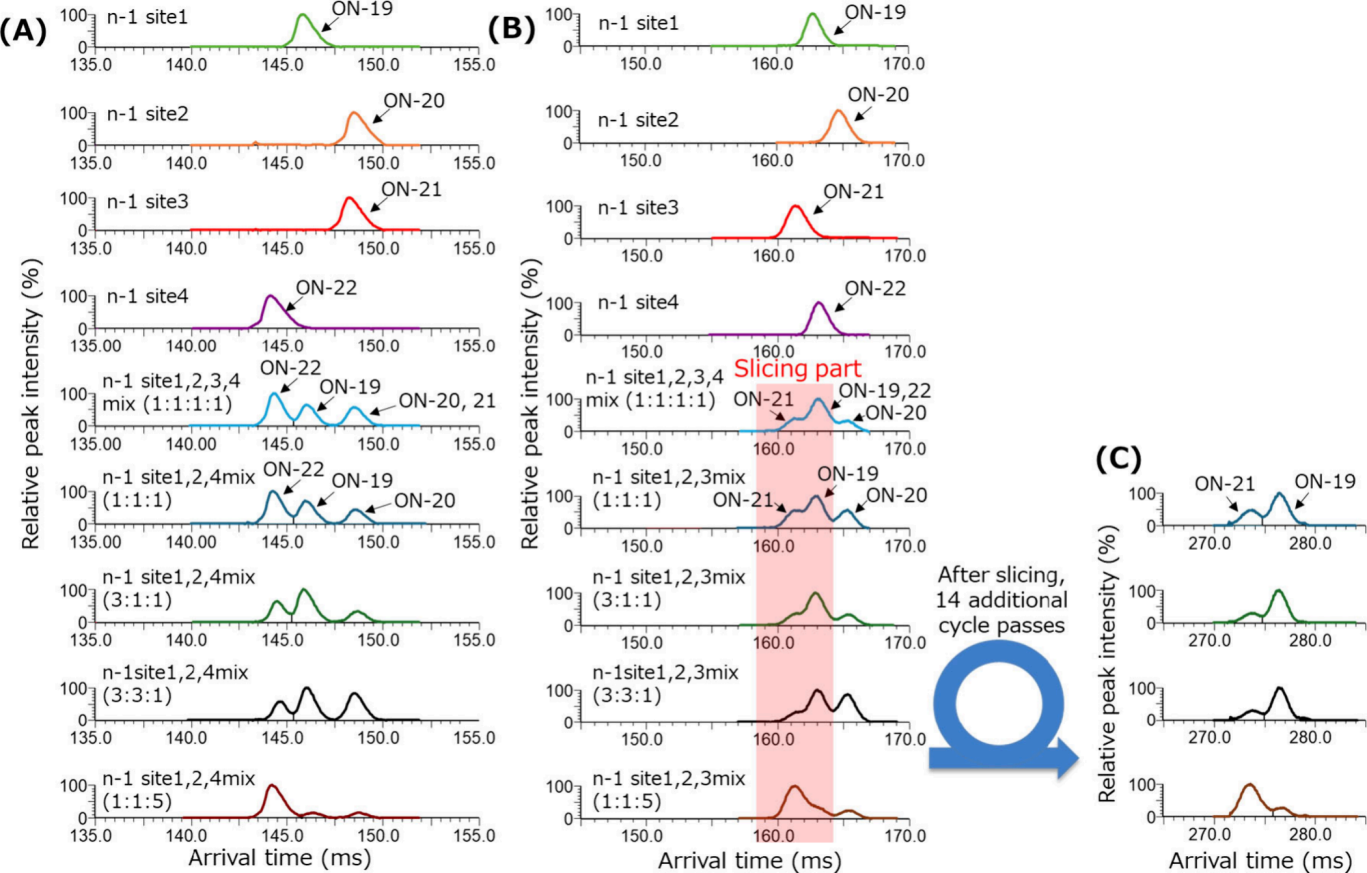
Mobiligrams of the *n* – 1 impurity isomers of the patisiran antisense strand: (A) Mobiligrams of the patisiran antisense strand *n* – 1 impurity isomers acquired at the 9– charge state by 21 cycles passed. (B) Mobiligrams of the patisiran antisense strand *n* – 1 impurity isomers acquired at the 8– charge state by 20 cycles passed. (C) Mobiligrams obtained after slicing the red-highlighted part.

We also evaluated the separation of four *n* – 1 impurity isomers of the patisiran antisense strand (ON-19–22). The mobiligrams of four *n* – 1 isomers of the patisiran antisense strand are shown in Figure 5. As with the case of abasic isomers, the charge state that enables the separation of all four isomers was not determined; however, it was observed that up to three isomers could be separated at 9– and 8– charge states. At the 9– charge state, *n* – 1 sites 1 and 4 were separated from each other, but the peaks of *n* – 1 sites 2 and 3 were not separated (Figure 5A). In contrast, at the 8– charge state, the peak of *n* – 1 site 2 was separated from those of the other three species, but the peaks of *n* – 1 sites 1 and 4 were completely overlapped. Moreover, the separation of the *n* – 1 site 3 was incomplete (Figure 5B). To improve the insufficient separation of the peaks, the slicing technique was applied, and an additional 14 cycle passes were performed (Figure 5C). Here, the % relative responses of the *n* – 1 site 1, 2, and 4 mixture at the 9– charge state and *n* – 1 site 1, 2, and 3 mixture at the 8– charge state were obtained. The % relative response results are shown in Table S3. After evaluating the results of the 9– charge state for *n* – 1 sites 1, 2 and 4, it was observed that the overall % relative responses of *n* – 1 site 2 tended to be low, and the lowest % relative response obtained was 68.4%. In contrast, in the results of the 8– charge state for *n* – 1 sites 1, 2, and 3, the % relative response of the result of mixing at 1:1:1 for the *n* – 1 site 2 was 61.7%, and the result of the mixing ratio of 3:3:1 for the *n* – 1 site 3 was 131.4%. These results confirmed that the separation of oligonucleotide isomer impurities was possible using cyclic IMS. Notably, oligonucleotide ions exhibit broad charge state distributions, and we confirmed that the mobiligram patterns drastically changed at each charge state. Based on these points, we confirmed that the most appropriate charge state for oligonucleotide isomer separation can be selected by acquiring several charge state mobiligrams. Our results suggested that ion mobility mass spectrometry can be applied to therapeutic oligonucleotide samples containing coeluting impurities.

### Coeluting FLP Sequence Interference against Isomer Impurity Mobiligrams

To simulate the analysis of these coeluting impurities in a real therapeutic oligonucleotide sample, the impurity standards needed to be mixed with the full-length product (FLP). Here, we analyzed 0.1 nmol/μL patisiran impurity isomer with and without 10 nmol/μL of FLP to verify if the excess FLP alters the mobiligram patterns. The abasic impurity mixture was spiked with the patisiran FLP antisense strand, and the *n* – 1 impurity mixture was spiked with the patisiran FLP sense strand. Both these results are shown in Figure S2. Under these conditions, the intensity of the isomer peaks decreased due to the ion suppression effect; however, both spiked samples showed equivalent mobiligram patterns. Although the oligonucleotide therapeutic active pharmaceutical ingredient typically has a complex impurity profile and a wide range of impurity levels, our results suggest that the cyclic IMS mobiligram is effective for analyzing oligonucleotide isomer impurities.

### Separation of Potential PS → PO Impurity Isomers of siRNA Givosiran by Cyclic IMS

**Figure 6:**
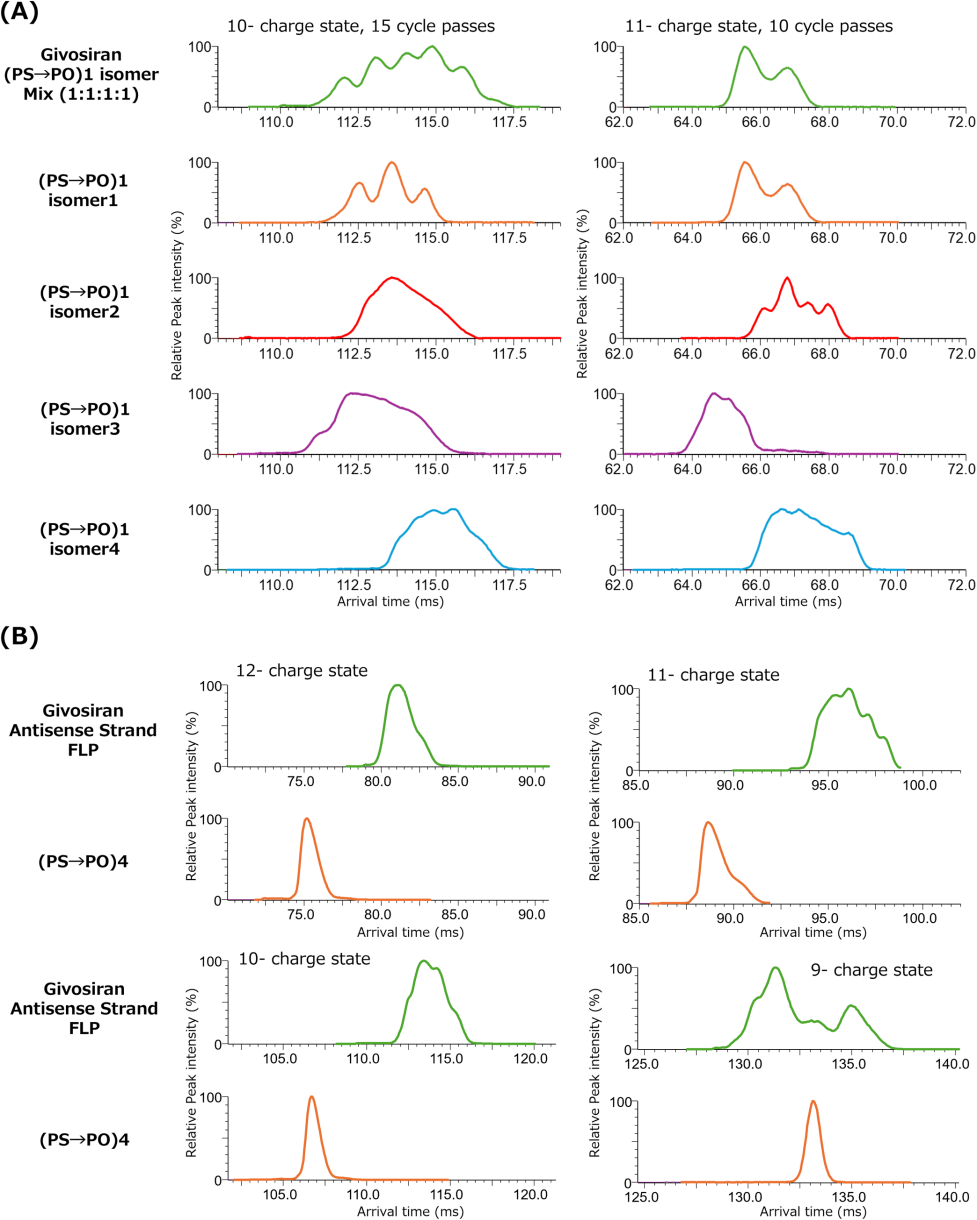
Mobiligrams of the (PS → PO) impurity isomers of the givosiran antisense strand: (A) Mobiligrams of the givosiran antisense (PS → PO)1 isomers and mixture acquired at the 10– charge state acquired by 15 cycle passes and the 11– charge state acquired by 10 cycle passes. (B) Mobiligrams of the givosiran antisense strand and (PS → PO)4 impurity acquired at 9– to 12– charge state by 15 cycle passes.

As it was shown in Figure 2, PS modification highly affects the mobiligram complexity. Therefore, we next selected a partially PS-modified siRNA, the givosiran antisense strand, as a model sequence to confirm if the PS → PO impurity isomers can be analyzed by cyclic IMS. The givosiran antisense contains four PS modifications within its sequence; therefore, we prepared multiple samples with different degrees of PS → PO linkages so that we could comprehensively evaluate how this structural change and the presence of diastereomers affect the mobiligrams. The givosiran impurity model sequences are also shown in Table 1. First, sequences ON-36–39 containing three PS linkages, described as (PS → PO)1, and their mixtures were measured. The mobiligrams of the 10– and 11– charge states are shown in Figure 6A. Multiple peak tops were observed for the mixed sample at both charge states, and broader mobiligrams were obtained compared to those obtained for PS-unmodified patisiran sense and antisense sequences (ON-9–22). Since the mobiligrams of each impurity isomer are complicated, it is difficult to analyze the mixing ratio. However, each isomer showed a specific mobiligram, and it was possible to distinguish the isomeric structures by cyclic IMS. Roussis et al. used metal ion complexation chromatography, reversed phase-strong anion exchange chromatography, and ^31^P nuclear magnetic resonance (NMR) to characterize the PS diastereomer profile.^27^ We consider that our cyclic IMS method is also applicable to determine the distribution of the diastereomers.

Next, three of the four PS-modified structures on the givosiran antisense strand were converted into PO structures ((PS → PO)3 isomers), and these samples were analyzed by cyclic IMS. Figure S3 shows the mobiligrams of these samples acquired by 15 cycles passed at the 10– charge state ions. As each sample contained only one PS-modified structure, they formed two diastereomers, the *S*-form and the *R*-form, and the two peaks separated from each other were confirmed. Here, (PS → PO)3 isomer 3 was only partially separated after 15 cycles, and an additional 20 cycles were performed to achieve the separation. From these results, we confirmed that oligonucleotide diastereomers produced from one PS modification can be separated, and the multiple PS-modified sequences are combined to form a broad peak shape.

Finally, the mobiligrams of the givosiran antisense strand and all its PO counterparts, (PS → PO)4, were compared. Figure 6B shows the mobiligrams of 9– to 12– charge state ions obtained by 15 cycle passes. It was confirmed that the target sequence containing four PS modifications showed a broad peak at any charge state compared to (PS → PO)4. The givosiran FLP contains 2^4^ = 16 diastereomers. Similar to any other analytical method such as HPLC, achieving the separation of each diastereomer by cyclic IMS was difficult. However, our results suggest that the givosiran diastereomer patterns can be compared by confirming mobiligram patterns as each complicated peak pattern is affected by diastereomer compositions.

## Conclusion

In this study, we used cyclic IMS, which is capable of high-resolution IM-MS measurement, to separate the potential *n* – 1 and abasic impurity isomers of the patisiran sense and antisense strands and the potential PS → PO impurity isomers of the givosiran antisense strand.

First, a feasibility study for cyclic IMS resolution was conducted using 8 mer short-sequence isomers with partial modifications. Next, the mobiligrams of all separated isomers were obtained except for PS containing sequences. We used four groups of patisiran impurity isomers as model sequences (a combination of the sense strand, the *n* – 1 form of the antisense strand, and the abasic form of patisiran). We discovered that it is possible to separate up to three isomers by selecting the appropriate charge state. We also confirmed that the mobiligram patterns of the impurity isomers did not change even in the presence of a large excess of the FLP sequence.

Next, we focused on the PS → PO impurities, which are known potential impurities of therapeutic oligonucleotides. The givosiran antisense strand, which contains four PS modifications, was used as a model, and the measurements were carried out on multiple sequences with different degrees of PS → PO replacements. Consequently, the analysis results of the impurity sample, in which one of the four PS modifications was changed to PO, showed a specific mobiligram that could be distinguished from other samples, although it was difficult to separate and characterize each impurity isomer ratio as in the case of full PO sequence impurity isomers. Additionally, from the analysis results of one PS modification remaining sample, two separated diastereomer peaks of the *S*-form and *R*-form were confirmed. Therefore, it is revealed that the combination of these diastereomeric peaks contributed to form a broad mobiligram peak shape.

Based on the results of this study, we concluded that cyclic IMS enables the separation and characterization of isomer impurities, which is difficult using LC/MS analysis. We believe that cyclic IMS technology will contribute to the analysis of therapeutic oligonucleotides.

## Supporting Information Available

Oligonucleotide structures; FLP sequence and impurity isomer spiked mass spectrum and mobiligram comparison; mobiligrams of givosiran antisense strand (PS → PO)1 impurity isomers; percent relative responses of impurity isomers of the patisiran sense and antinsense strands (PDF)
