# Supplementary material for: Separation and Characterization of Therapeutic Oligonucleotide Isomer Impurities by Cyclic Ion Mobility Mass Spectrometry
Source: J Am Soc Mass Spectrom. 2024 Jul 31;35(9):2156–64. doi: 10.1021/jasms.4c00197 (PMC11378280; doi:10.1021/jasms.4c00197)
Supplement: Supplementary file 1 — js4c00197_si_001.pdf [file js4c00197_si_001.pdf]

Supporting information

Separation and characterization of therapeutic oligonucleotide isomer impurities by cyclic ion mobility-mass spectrometry

Shogo Omuro[1]; Takao Yamaguchi[1]; Taiji Kawase[2]; Kenji Hirose[2]; Tokuyuki Yoshida[3]; Takao Inoue[3]; Satoshi Obika[1]*

[1] Graduate School of Pharmaceutical Sciences, Osaka University, 1-6 Yamadaoka, Suita, Osaka, 565-0871, Japan.
[2] Nihon Waters KK, Kitashinagawa,Shinagawa, Tokyo, 140-0001, Japan.
[3] Division of Molecular Target and Gene Therapy Products, National Institute of Health Sciences, 3-25-26 Tonomachi, Kawasaki-ku, Kawasaki, Kanagawa, 210-9501, Japan.

*To whom correspondence should be addressed

Tel: +81-6-6879-8200 Fax: +81-6-6879-8204
E-mail: obika@phs.osaka-u.ac.jp

Table of Contents:

Figure S1 Oligonucleotide structures related in this study S-2

Figure S2 FLP sequence and impurity isomer spiked mass spectrum and mobiligram comparison... S-3

Figure S3 Mobiligrams of givosiran antisense strand (PS→PO)1 impurity isomers S-4

Table S1 Percent relative responses of the n−1 and abasic impurity isomers of the patisiran sense strand in the 10- charge state mobiligrams S-5

Table S2 Percent relative responses of the abasic impurity isomers of the patisiran antisense strand S-5

Table S3 Percent relative responses of patisiran antisense strand n−1 impurity isomers........ S-6

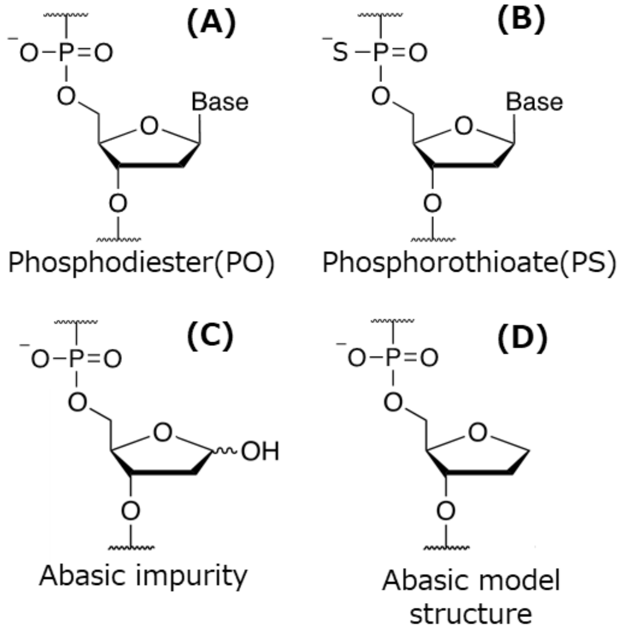

Figure S1 Oligonucleotide structures related in this study

(A) Phosphodiester structure in the DNA strand; (B) Modification of the PS linkage, wherein the non-bridging oxygen atom is replaced by a sulfur atom; (C) Abasic structure observed in therapeutic oligonucleotide impurities; (D) 1',2'-Dideoxyribose abasic structure used in this study.

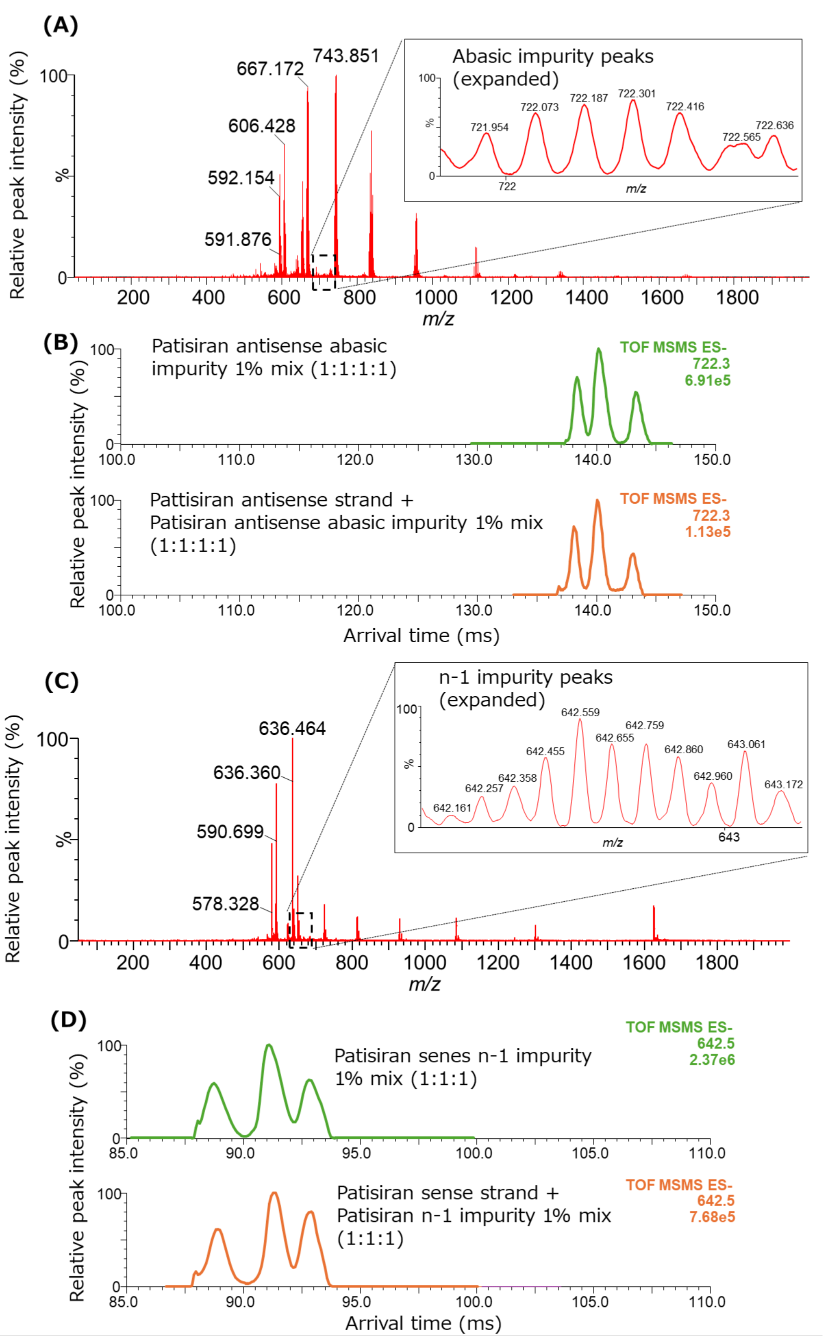

Figure S2 FLP sequence and impurity isomer spiked mass spectrum and mobiligram comparison

(A) The 9− charge state mass spectrum of the patisiran antisense sequence (10 nmol/μL) added to the 0.1 nmol/μL abasic isomer mixture (1:1:1:1). (B) Comparison of mobiligrams obtained with and without the patisiran antisense sequence. (C) The 10− charge state mass spectrum of the patisiran sense sequence (10 nmol/μL) added to the 0.1 nmol/μL n−1 isomer mixture (1:1:1). (D) Comparison of mobiligrams obtained with and without the patisiran sense sequence.

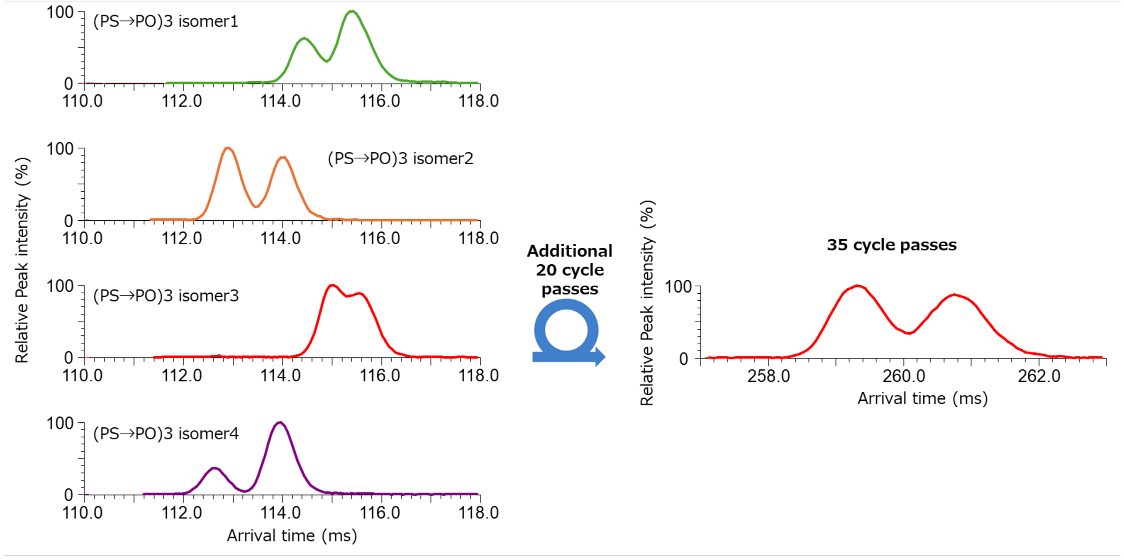

Figure S3 Mobiligrams of givosiran antisense strand (PS→PO)3 impurity isomers

(Left) Mobiligrams of the givosiran antisense (PS→PO)3 isomers acquired at the 10− charge state by 15 cycle passes. (Right) Mobiligrams of the givosiran antisense (PS→PO)3 isomers acquired after additional 20 cycle passes.

Table S1 Percent relative responses of the n−1 and abasic impurity isomers of the patisiran sense strand in the 10− charge state mobiligrams

	Mixture ratio (Site1,2,3)		Standard	1:1:1	3:1:1	3:3:1	1:1:5
n−1 isomer	n−1 site1 (ON-9)	Peak Area	445429	222777	212406	164435	65283
		% relative response	-	90.9	89.3	85.4	103.2
	n−1 site2 (ON-10)	Peak Area	349886	226359	84344	172268	70550
		% relative response	-	117.6	135.5	113.9	142.0
	n−1 site3 (ON-11)	Peak Area	858069	431804	147460	126297	554129
		% relative response	-	91.5	96.6	102.1	91.0
Abasic isomer	Abasic site1 (ON-12)	Peak Area	233624	50633	163231	130422	56154
		% relative response	-	99.3	97.6	93.1	130.4
	Abasic site2 (ON-13)	Peak Area	228274	134904	135950	115038	50636
		% relative response	-	117.8	113.4	103.2	166.8
	Abasic site3 (ON-14)	Peak Area	265474	109582	120678	38983	152911
		% relative response	-	82.2	86.6	90.3	86.6

Table S2 Percent relative responses of the abasic impurity isomers of the patisiran antisense strand

	Mixture ratio		Standard	1:1:1	3:1:1	3:3:1	1:1:5
9− charge State (site2,3,4)	Abasic site2 (ON-16)	Peak Area	886138	99564	676172	385407	158087
		% relative response	-	116.6	108.9	113.4	115.2
	Abasic site3 (ON-17)	Peak Area	1172704	100138	232992	393406	185468
		% relative response	-	88.6	85.0	87.5	102.2
	Abasic site4 (ON-18)	Peak Area	1302352	118838	269053	161898	972987
		% relative response	-	94.7	88.4	97.3	96.5
8− charge State (site1,2,4)	Abasic site1 (ON-15)	Peak Area	452888	150733	306272	171852	31678
		% relative response	-	99.7	104.6	111.8	81.5
	Abasic site2 (ON-16)	Peak Area	500351	141469	91469	145422	35608
		% relative response	-	84.7	84.8	85.7	82.9
	Abasic site4 (ON-18)	Peak Area	432888	166866	94575	52642	198937
		% relative response	-	115.5	101.4	107.5	107.1

Table S3 Percent relative response of patisiran antisense strand n−1 impurity isomers

		Mixture ratio	Standard	1:1:1	3:1:1	3:3:1	1:1:5
9− charge State	n−1 site1 (ON-19)	Peak Area	879377	404786	500940	361456	106178
		% relative response	-	119.8	101.3	109.5	101.8
	n−1 site2 (ON-20)	Peak Area	958593	251915	164208	318969	107796
		% relative response	-	68.4	91.4	88.6	94.8
	n−1 site4 (ON-22)	Peak Area	1347338	578160	264152	178030	804500
		% relative response	-	111.7	104.6	105.6	100.7
8− charge State	n−1 site1 (ON-19)	Peak Area	802010	228832	272222	206497	71916
		% relative response	-	112.8	90.6	89.5	65.2
	n−1 site2 (ON-20)	Peak Area	1339619	182646	228454	376323	115067
		% relative response	-	61.7	67.4	96.1	88.7
	n−1 site3 (ON-21)	Peak Area	510323	112538	81579	64303	375030
		% relative response	-	87.2	128.1	131.4	107.0
